# Editorial: Health and psychological adaptations to life challenges and stressful conditions

**DOI:** 10.3389/fpsyg.2025.1691571

**Published:** 2025-09-26

**Authors:** Iuliia Pavlova, Aleksandra Maria Rogowska, Ann T. Skinner, Anton Kurapov

**Affiliations:** ^1^Lviv State University of Physical Culture, Lviv, Ukraine; ^2^Helsinki Collegium for Advanced Studies, University of Helsinki, Helsinki, Finland; ^3^Faculty of Social Sciences, Institute of Psychology, University of Opole, Opole, Poland; ^4^Center for Child and Family Policy, Duke University, Durham, NC, United States; ^5^Department of Psychology, Faculty of Natural Sciences, Centre for Cognitive Neuroscience, University of Salzburg, Salzburg, Austria; ^6^Faculty of Psychology, Taras Shevchenko National University of Kyiv, Kyiv, Ukraine

**Keywords:** coping, resilience, stress, mental health, wellbeing, resources, war, pandemic

## Introduction

Adaptation to adversity is a multifaceted process shaped by individual resilience, social support, coping strategies, and environmental context. The capacity to adapt to life challenges is a defining feature of human resilience, encompassing psychological, social, and biological mechanisms that sustain functioning under stress. This Research Topic in *Frontiers in Psychology*, and *Frontiers in Public Health* gathers empirical evidence from diverse populations experiencing acute and chronic stressors, including armed conflict, pandemics, relocation stress, occupational trauma, and mass-casualty events.

The 11 peer-reviewed articles in this Research Topic employ cross-sectional, longitudinal, and mixed-method designs to identify patterns of distress, highlight protective factors, and inform tailored interventions. The manuscripts represent work from multiple cultural and geographical contexts including Canada, China, Israel, Portugal, Singapore, Switzerland, Ukraine, and multinational expatriate populations. Different perspectives on the mechanisms underpinning adaptation to life challenges are presented through the Research Topic. Collectively, these studies illustrate how individuals and professionals utilize coping strategies, leverage social and psychological resources, and negotiate long-term health consequences in high-adversity environments. They also underscore the variability of adaptation trajectories, calling for context-sensitive interventions and sustained monitoring to protect and enhance wellbeing. The aim of this editorial is to synthesize the key insights from the included studies, place them within the broader landscape of adaptation research, and highlight implications for clinical practice, public health, and community-based interventions.

## Civilian adaptation under armed conflict

Armed conflicts exert profound and often prolonged effects on civilian mental health. The psychological toll is shaped not only by direct exposure to violence but also by displacement, loss of livelihood, and ongoing uncertainty. Research in this area seeks to quantify mental health impacts, identify protective factors, and inform targeted interventions in affected populations.

Kurapov et al. report on a cross-sectional study of Ukrainian civilians during the Russian–Ukrainian war, assessing PTSD, depression, anxiety, sleep disturbance, prolonged grief, and resilience. The sample, drawn from regions with varying exposure to hostilities, showed high comorbidity among individuals directly affected by shelling, occupation, or displacement. Diamant and Kalfon Hakhmigari surveyed employed Israeli civilians 1 month after the onset of war, and showed associations between temporal disorientation and higher work–family conflict, emotional distress, and burnout. Despite limited direct injury exposure, many participants reported emotional strain yet also reported elements of post-traumatic growth, highlighting the dual presence of vulnerability and resilience in shared-trauma contexts. The findings underscore the need for sustained psychosocial monitoring and targeted, context-sensitive interventions.

## Occupational stress and coping in prolonged crises

Workers in healthcare and other high-stress sectors face unique challenges when crises are prolonged. Beyond acute stress reactions, they may experience chronic distress, burnout, or delayed onset of mental health symptoms.

Coleman et al. conducted a longitudinal study of Canadian healthcare providers during the COVID-19 pandemic, identifying five mental health trajectories: resilient, chronically distressed, delayed onset, recovery, and mutable. Chronically distressed participants were nearly seven times more likely to meet PTSD criteria than resilient peers, pointing to the need for early identification of PTSD symptomology and intervention. Jin et al. studied Chinese clinical nurses and found that coping strategies mediated the relationship between occupational stress and mental health. Adaptive coping (e.g., problem-solving) buffered negative impacts, whereas maladaptive coping (e.g., avoidance) exacerbated them. Zhang et al. examined ICU nurses and showed that psychological capital appreciation fully mediated the resilience–burnout relationship, accounting for 79% of the total effect. Enhancing hope, optimism, self-efficacy, and resilience emerged as a promising prevention strategy. Gunasekaran et al. assessed resilience among Singapore residents during COVID-19 and found normal overall levels but significantly lower scores among individuals experiencing anxiety, depression, or job insecurity. Together, all these findings support routine trajectory monitoring, early PTSD-risk detection, and interventions that build adaptive coping and psychological capital while addressing socioeconomic stressors.

## Professional resilience and secondary trauma

Professionals providing care or aid in high-trauma environments may be doubly impacted—by their own exposure to crisis and by absorbing the trauma of those they support. Such increased exposure can lead to vicarious trauma but may also foster growth and resilience.

George-Levi et al. examined Israeli trauma therapists working in the aftermath of the October 7 attack and found that hope acted as a consistent stress buffer across all levels of secondary trauma. This reinforces the role of hope as a critical professional resilience factor in shared traumatic reality contexts, complementing the growth processes described by Diamant and Kalfon Hakhmigari. Coelho et al. extend this theme to palliative care professionals, highlighting occupational resilience challenges in end-of-life contexts. Their scoping review shows that early bereavement risk assessment, structured communication protocols, and training to detect complicated grief are not only critical for caregiver families but also serve as protective measures for staff wellbeing. By ensuring referral pathways and fostering proactive emotional support systems, organizations can mitigate secondary trauma and strengthen professional resilience. Overall, the findings favor paired person-focused (hope-building, reflective practice) and system-focused (protocolized bereavement support, supervision/peer consultation) strategies to protect helpers while preserving quality of care.

## Psychological resources and social support as buffers

Psychological resources such as optimism and self-efficacy, combined with strong social support networks, can buffer individuals from the full psychological impact of severe stress. These factors can operate at individual, interpersonal, and community levels. Across the included studies, individual emotion regulation and sleep-linked affect (Chen et al.; Xiao et al.), hopeful/proactive coping and perceived support during relocation (Aegerter et al.) converge on a common pattern: resources reduce distress when they are matched to exposure and situated within supportive systems. Meaning in work protects only at low-to-moderate secondary trauma, whereas hope buffers stress across exposure levels (George-Levi et al.), and structured bereavement guidance operationalizes caregiver support (Coelho et al.). Taken together, these findings support multi-level interventions that strengthen sleep and emotion regulation, deliberately cultivate hope, and institutionalize culturally responsive support pathways calibrated to trauma severity.

## Dynamic nature of adaptation

Adaptation is not a fixed state but a fluid process that evolves over time. Trajectories of recovery, stability, and decline can shift depending on ongoing exposures, resource availability, and personal circumstances.

Within the healthcare worker population, varying responses are seen, including resilience, delayed distress and recovery, but also chronic distress (Coleman et al.). Similar resilience variability is also seen among civilians in war zones (Kurapov et al.) and in palliative care environments (Coelho et al.). Together, these studies suggest that even across varying contexts, the adaptation trajectories over time and variability in response follow patterns that can be predicted by a similar set of factors including early monitoring, early intervention, sustained social and therapeutic support, pre-loss/trauma preparation, and post-loss/trauma follow-up.

## Implications and future directions

The synthesis of these studies demonstrates that adaptation to life challenges is a dynamic, context-dependent process that benefits from early detection of distress, continuous monitoring of resilience, and the promotion of psychological and social resources. This Research Topic shows the need for tailored interventions and flexible supports that adapt to changing needs. Programs that combine skill-building at the individual level with organizational and community engagement appear especially promising. The studies also emphasize the need for culturally sensitive approaches that reflect local contexts while drawing on best practices from international and clinical experience. Moving forward, longitudinal and adaptive research designs will be essential for tracking changes in resilience, understanding how protective factors evolve, and refining strategies to strengthen health and psychological adaptation in the face of ongoing or recurring adversity.

